# Identification of circRNA‐disease associations via multi‐model fusion and ensemble learning

**DOI:** 10.1111/jcmm.18180

**Published:** 2024-03-20

**Authors:** Jing Yang, Xiujuan Lei, Fa Zhang

**Affiliations:** ^1^ School of Computer Science Shaanxi Normal University Xi'an Shaanxi China; ^2^ School of Medical Technology Beijing Institute of Technology Beijing China

**Keywords:** attention mechanism, CircRNA‐disease association, ensemble learning, metapath

## Abstract

Circular RNA (circRNA) is a common non‐coding RNA and plays an important role in the diagnosis and therapy of human diseases, circRNA‐disease associations prediction based on computational methods can provide a new way for better clinical diagnosis. In this article, we proposed a novel method for circRNA‐disease associations prediction based on ensemble learning, named ELCDA. First, the association heterogeneous network was constructed via collecting multiple information of circRNAs and diseases, and multiple similarity measures are adopted here, then, we use metapath, matrix factorization and GraphSAGE‐based models to extract features of nodes from different views, the final comprehensive features of circRNAs and diseases via ensemble learning, finally, a soft voting ensemble strategy is used to integrate the predicted results of all classifier. The performance of ELCDA is evaluated by fivefold cross‐validation and compare with other state‐of‐the‐art methods, the experimental results show that ELCDA is outperformance than others. Furthermore, three common diseases are used as case studies, which also demonstrate that ELCDA is an effective method for predicting circRNA‐disease associations.

## 1 INTRODUCTION

Circular RNAs (circRNAs) are a class of non‐coding RNA with a closed structure, and there is accumulating evidence indicating that circRNA plays an important role in biological processes such as the genetic aetiology of human complex diseases.^1^ circRNA was first observed in the cytoplasm of eukaryotic cells.^2^ In the past, limited by technology, the research on circRNA has not been well‐developed, but in recent years, high‐throughput sequencing technologies have developed rapidly, the amount of circRNAs appears an exponential growth trend, and multiple databases of circRNAs have been established. CircBase,^3^ which collects information of circRNAs on multiple species; circBank,^4^ a comprehensive database of more than 140,000 human annotated circRNAs, based on the data of all humans in circBase database, further analysis and processing were conducted, which also provides miRNA‐circRNA interactions; circNet,^5^ an updated database for exploring circular RNA regulatory networks in cancers; circFunBase,^6^ a web‐accessible database that can provide a high‐quality functional circRNA resource; exoRBase^7^ provides the comprehensive annotation and expression landscapes of circRNAs; and circR2Disease,^8^, ^9^ circRNADisease,^10^ and circ2Disease v2.0^11^ are databases that manually curated experiment‐supported human circRNAs related to diseases.

As a result, identifying potential circRNA‐disease associations via wet‐lab experiment is time‐consuming and costly, which urge researchers to explore effective computational methods based on known associations and biological information.^12^ These methods can be roughly divided into two categories: traditional machine learning‐based methods and deep learning‐based methods.

Traditional machine learning‐based methods always treat the association prediction problem as a binary classification problem. Fan et al.^13^ proposed a method (KATZHCDA) based on KATZ measure for predicting unknown circRNA‐disease associations; however, the network structure has a significant impact on model performance. Zhao et al.^14^ proposed a computational method IBNPKATZ, which also base on KATZ measurement, heavily relies on the structure of network. Yan et al.^15^ developed a method (DWNN‐RLS) based on Regularized Least Squares (RLS) of Kronecker product kernel and Decreasing Weight K‐Nearest Neighbour (DWNN), due to the calculation process of Kronecker product, it is not suitable for large‐scale datasets. Wei et al.^16^ proposed a novel computational method (icircDA‐MF) based on Matrix Factorization (MF), which introduced the information of gene in this work. Zhao et al.^17^ proposed a method based on locality‐constrained linear coding, but the calculation of circRNAs and diseases similarity matrices will lead some bias. Peng et al.^18^ proposed a method (RNMFLP) combining Robust Nonnegative Matrix Factorization (RNMF) and Label Propagation (LP). Wang et al.^19^ developed a method (KNN‐NMF) using weighted K nearest neighbours to reduce the false‐negative association impact on prediction performance; however, the construction of similarity networks for the above three models are only depending on the topology information and ignoring the biological attribute information. Zhang et al.^20^ predicted associations via metapath2vec++ and matrix factorization, metapath2vec++ requires prior specification of metapaths and inefficient for large‐scale networks. Ding et al.^21^ predicted associations based on variational graph autoencoder with matrix factorization, where the variational auto‐encoder assumes the latent variable follows a simple gaussian distribution, limits the expressiveness of the learned embeddings. Zhang et al.^22^ proposed a novel method (ICDMOE) for predicting circRNA‐disease associations through a multi‐objective evolutionary algorithm, but the interaction of features is not considered in the model.

Deep learning‐based methods usually learn feature embeddings of circRNAs and diseases on neural networks. Wang et al.^23^ developed a method (GCNCDA) based on multi‐similarity fusion and Fast learning with Graph Convolutional Network (FastGCN), where GCN is sensitivity to graph structures and has limited generalization capability. Bian et al.^24^ proposed a method (GATCDA) to predict circRNA‐disease associations based on graph attention network, and the performance of the model highly depended on the attention mechanism, requiring careful tuning and optimization. Zheng et al.^25^ develop a method (iCDA‐CGR) based on Chaos game representation to identify circRNA‐disease associations, where Chaos game needs a large number of iterations for obtain the expressive representations. Ji et al.^26^ proposed a method (GATNNCDA) that combines Graph Attention Network (GAT) and multi‐layer neural network to infer disease‐related circRNAs, but the similarity network of circRNAs is highly dependent on the circRNA‐disease network. Wang et al.^27^ predicted unknown associations based on GraRep, where GraRep proposed only for homogeneous graphs, and the performance on heterogeneous graphs is limited. Chen et al.^28^ proposed a novel method via signed heterogeneous graph network, due to the computational complexity, it is not suitable for large‐scale graphs. Chen et al.^29^ proposed a method (RGCNCDA) based on Relational Graph Convolutional Network (RGCN) and incorporate microRNA (miRNA) to improve the prediction performance, however, RGCN mainly focuses on the local information, and ignores the global information. Guo et al.^30^ proposed a method (THGNCDA) using graph neural network with attention to learn the importance of its each neighbour, but the model complexity is relatively high.

We propose a novel Ensemble Learning‐based CircRNA‐Disease Association prediction method (short for ELCDA) in this work. First, a heterogeneous network is constructed and multiple similarities are calculated based on different views; then, MAGNN (metapath aggregated graph neural network),^31^ CMF^32^ and GraphSAGE^33^ are used to obtain the comprehensive representations of circRNAs and diseases; and the embeddings obtained by different models are fed into different classifiers, a soft voting strategy is used to fuse the classification results and obtain the final prediction results.

In summary, the main contributions of this study are listed as follows:

1. A 3‐layer heterogenous network is constructed among circRNA, miRNA and disease, and 4 different similarity measurements are calculated from multi‐views;
2. The metapath‐based feature extractor mainly used to capture global information, GraphSAGE is used to obtain the local, nonlinear features, linear information is obtained via MF, the comprehensive representation can be obtained by integrating these features together;
3. Multiple classifiers are used here, and then an ensemble learning method is adopted to obtain the final predicted results.

## 2 MATERIALS AND METHODS

### 2.1 Problem description

**FIGURE 1:**
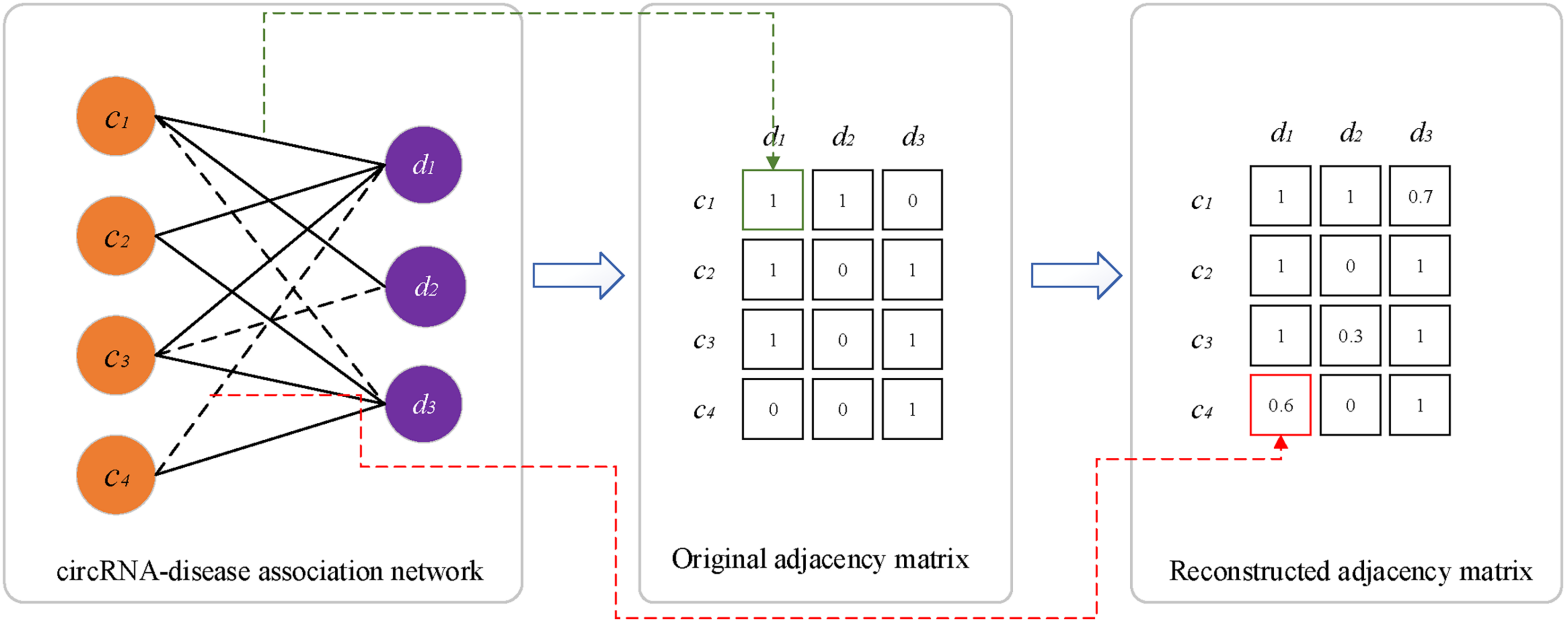
The circRNA‐disease association prediction problem.

The network of circRNA‐disease associations can be considered as a bipartite network, assuming there are *m* circRNAs and *n* diseases in the network, the nodes can be denoted as two sets $T_{C}=\left\{c_{1},c_{2},\cdots ,c_{m}\right\}$ and $T_{D}=\left\{d_{1},d_{2},\cdots ,d_{n}\right\}$, and there are three types of edges between nodes, which can be denoted as $E=\left\{e_{\mathrm{cc}},e_{\mathrm{cd}},e_{\mathrm{dd}}\right\}$, where $e_{\mathrm{cc}}$ and $e_{\mathrm{dd}}$ are the similarity between circRNAs and diseases, $e_{\mathrm{cd}}$ is the association between circRNA and disease, if circRNA *c* is related to disease *d*, $e_{\mathrm{cd}}=1$, else, $e_{\mathrm{cd}}=0$. The goal of our study was to reconstruct the adjacency matrix between circRNAs and diseases and make it as similar as possible to the original adjacency matrix, the values greater than 0 in the reconstructed matrix demonstrate that the corresponding circRNAs and diseases may have associations. As shown in Figure 1, the black solid lines and black dashed lines represented the known associations and predicted associations, respectively.

### 2.2 Materials

In this paper, we collect the information of circRNAs, miRNAs and diseases, the known associations among circRNAs, miRNAs and diseases are downloaded from circBank, circR2Disease V2.0 and HMDD V3.2,^34^ after data preprocessing, we obtain a dataset which contains 2223 circRNAs, 996 miRNAs and 199 diseases, the details are shown in Table 1.

**TABLE 1:** Basic information of dataset.

Types	Items	Numbers	Resources
Node	circRNA (C)	2223	circR2Disease, exoRBase
	Disease (D)	199	circR2Disease, MeSH
	miRNA (M)	996	HMDD
Edge	C‐D	2970	circR2Disease
	C‐M	13,408	circBank
	M‐D	10,282	HMDD
Metapaths	CDC, CMC, CDMDC, CMDMC, DCD, DMD, DCMCD, DMCMD		

Furthermore, we analyse the distribution frequency of each type of association, as shown in Table 2, (A) the number of circRNA‐related diseases; (B) the number of disease‐related circRNAs; (C) the number of circRNA‐related miRNAs; (D) the number of miRNA‐related circRNAs; (E) the number of miRNA‐related diseases; (F) the number of disease‐related miRNAs. It can be seen that most circRNAs are only related to one disease (about 80%), which demonstrated that the adjacency matrix of circRNA‐disease heterogeneous network is very sparse.

**TABLE 2:** Frequency distribution of each type of association.

Numbers	Type					
	A	B	C	D	E	F
1	51 (25.6%)	1834 (82.5%)	1 (0.1%)	148 (6.7%)	1(0.1%)	1 (0.5%)
2–5	62 (31.2%)	359 (16.1%)	699 (70.2%)	1344 (60.5%)	594 (59.6%)	51 (25.6%)
6–10	29 (14.6%)	23 (1%)	15 (1.5%)	357 (16.1%)	109 (10.9%)	16 (8%)
11–50	43 (21.6%)	7 (0.3%)	178 (17.9%)	342 (15.4%)	265 (26.6%)	65 (32.7%)
>50	14 (7%)	0 (0%)	103 (10.3%)	32 (1.4%)	27 (2.7%)	66 (33.2%)
Total	199	2223	996	2223	996	199

**FIGURE 2:**
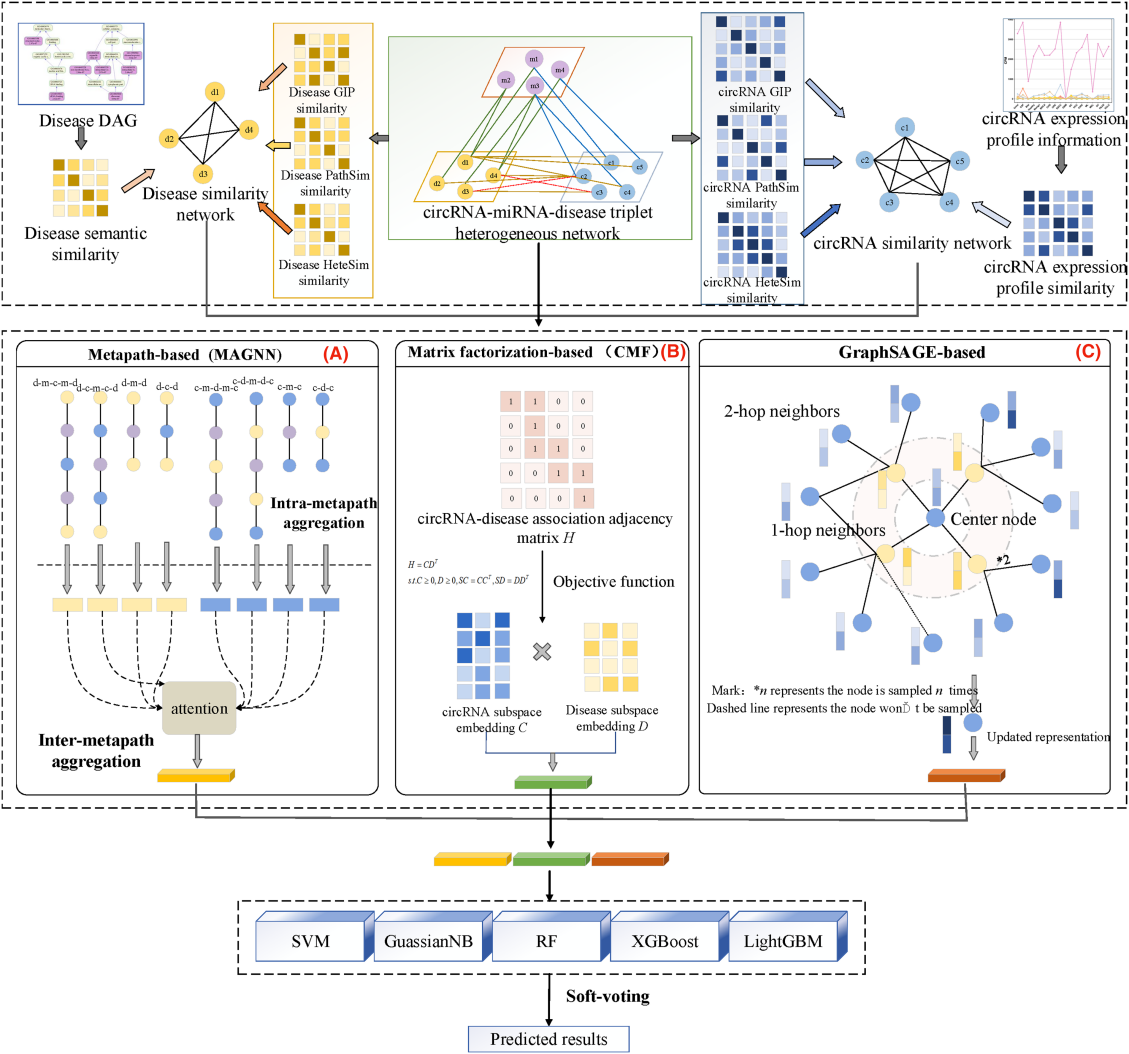
The flowchart of ELCDA.

The overview of proposed model is shown in Figure 2, which mainly consists of three modules: heterogeneous network construction, feature extraction and association prediction. Specifically, the high‐quality and sub‐structural features of nodes can be obtained via metapath‐based feature extractor, the low‐level and linear features of nodes can be obtained via matrix factorization (MF)‐based feature extractor, the local and nonlinear features can be obtained via GraphSAGE‐based feature extractor, then the ensemble learning is used to fusion them and obtain the classification results of unknown associations.

Based on the assumption that similar circRNAs are tend to related to similar diseases, several kinds of information are introduced to calculate the similarity matrices of circRNAs and diseases. In circRNA space, the expression profile similarity and functional similarity are used to build the circRNA similarity network, in disease space, the semantic similarity, gaussian interaction profile (GIP) kernel similarity are used to construct the disease similarity network, furthermore, *PathSim* and *HeteSim* are used here, the details are shown as follows:

Definition 1 Heterogeneous graph.^35^ A graph can be denoted as $G=\left(V,E\right)$, where *V* is the set of nodes and *E* is the set of edges. $\Gamma_{v}$ and $\Gamma_{e}$ are the sets of node types and edge types, respectively, where there are two mappings satisfying: and $\phi_{e}:e\to \Gamma_{e}$, if $|\Gamma_{v}|+|\Gamma_{e}|>2$, then *G* is a heterogeneous graph, otherwise, *G* is homogeneous.

Definition 2 Metapath.^36^ A metapath *P* is a special path that connects two entities in the form $o_{1}\overset{R_{1}}{\to }o_{2}\overset{R_{2}}{\to }\cdots \overset{R_{q-1}}{\to }o_{q}$, which can be abbreviated as $o_{1}o_{2}\cdots o_{q}$, $R=R_{1}\circ R_{2}\circ \cdots \circ R_{q-1}$ is the composite relation between start node $o_{1}$ and target node $o_{q}$, *q* is the length of path.

Definition 3 PathSim.^36^ Given a symmetric metapath *P*, the *PathSim* between two objects *x* and *y* of the same type is defined as follows:

(1) $$\text{PathSim}\left(x,y\right)=\frac{2\times \left|\left\{p_{x\to y}:p_{x\to y}\in P\right\}\right|}{\left|\left\{p_{x\to x}:p_{x\to x}\in P\right\}\right|+\left|\left\{p_{y\to y}:p_{y\to y}\in P\right\}\right|}$$

where $p_{x\to y}$ is a metapath instance from *x* to *y*.

Definition 4 HeteSim.^37^ Given a relevance path *P* corresponding to the relation *R* defined above, the *HeteSim* between two objects *x* and *y* is:

(2) $$\begin{array}{c} \text{HeteSim}\left(x,y|R\right)=\text{HeteSim}\left(x,y|R_{1}\circ R_{2}\circ \cdots \circ R_{l}\right)\\ =\frac{1}{\left|O\left(x|R_{1}\right)\right|\left|I\left(y|R_{l}\right)\right|}\sum_{i=1}^{\left|O\left(x|R_{1}\right)\right|}\sum_{j=1}^{\left|I\left(y|R_{l}\right)\right|}\text{HeteSim}\left(O_{i}\left(x|R_{1}\right),I_{j}\left(y|R_{l}\right)|R_{2}\circ \cdots \circ R_{l-1}\right) \end{array}$$

The *HeteSim* can be further simplified into the following form:

(3) $$\text{HeteSim}\left(x,y|P\right)=\frac{T_{P_{L}}\left(x,:\right){\left(T_{P_{R}^{-1}}\left(y,:\right)\right)}^{\text{Transpose}}}{{\left\|T_{P_{L}}\left(x,:\right)\right\|}_{2}{\left\|T_{P_{R}^{-1}}\left(y,:\right)\right\|}_{2}}$$

Assuming the middle node between *x* and *y* via path *P* is *mid*, then we can split *P* into $P_{L}=\left(x\cdots \mathrm{mid}\right)$ and $P_{R}=\left(\mathrm{mid}\cdots y\right)$, and *T* is the transition probability matrix, which can be calculated as:

(4) $$\begin{array}{c} T_{\mathrm{XY}}\left(x,y\right)=\frac{A_{\mathrm{XY}}\left(x,y\right)}{\sum_{k}A_{\mathrm{XY}}\left(x,k\right)} \end{array}$$

where *A*_*XY*_ is the adjacency matrix between node types *X* and *Y*.

**FIGURE 3:**
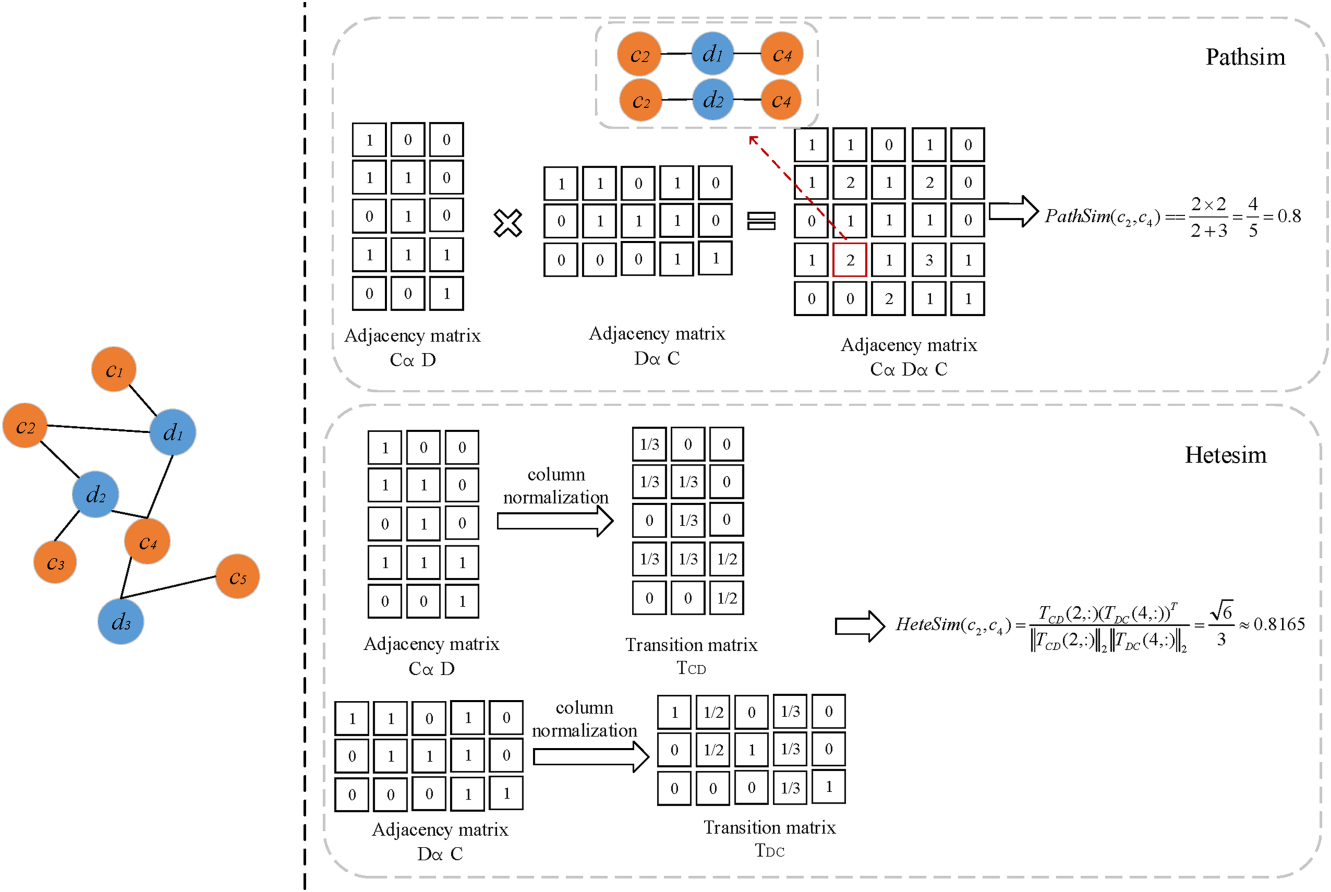
An example of Pathsim and Hetesim.

As shown in Figure 3, the details of calculating the *PathSim* and *HeteSim* score between *c*_*2*_ and *c*_*4*_ is shown as follows, we can see there are 2 kinds of path instances under path *P* = CDC between *c*_*2*_ and *c*_*4*_.

1. *PathSim*
$$\text{PathSim}\left(x,y\right)=\frac{2\times \left|\left\{p_{c_{2}\to c_{4}}:p_{c_{2}\to c_{4}}\in P\right\}\right|}{\left|\left\{p_{c_{2}\to c_{2}}:p_{c_{2}\to c_{2}}\in P\right\}\right|+\left|\left\{p_{c_{4}\to c_{4}}:p_{c_{4}\to c_{4}}\in P\right\}\right|}=\frac{2\times |\left\{c_{2}d_{1}c_{4},c_{2}d_{2}c_{4}\right\}|}{|\left\{c_{2}d_{1}c_{2},c_{2}d_{2}c_{2}\right\}|+|\left\{c_{4}d_{1}c_{4},c_{4}d_{2}c_{4},c_{4}d_{3}c_{4}\right\}|}=\frac{2\times 2}{2+3}=\frac{4}{5}=0.8$$
2. *HeteSim*

First, split *P* into *P*_*L*_ = CD and *P*_*R*_ = DC, then the adjacency matrices of *P*_*L*_ and *P*_*R*_ are denoted as *A* and *A* ^*Transpose*^, respectively, and obtain the transition matrices *T*_*CD*_ and *T*_*DC*_ via row normalization.

$$A=A_{\mathrm{CD}}=\left[\begin{array}{cc} \begin{array}{c} 1\\ 1\\ 0 \end{array} & \begin{array}{c} \begin{array}{cc} 0 & 0 \end{array}\\ \begin{array}{cc} 1 & 0 \end{array}\\ \begin{array}{cc} 1 & 0 \end{array} \end{array}\\ \begin{array}{c} 1\\ 0 \end{array} & \begin{array}{c} \begin{array}{cc} 1 & 1 \end{array}\\ \begin{array}{cc} 0 & 1 \end{array} \end{array} \end{array}\right],T_{\mathrm{CD}}=\left[\begin{array}{cc} \begin{array}{c} 1\\ 1/2\\ 0 \end{array} & \begin{array}{c} \begin{array}{cc} 0 & 0 \end{array}\\ \begin{array}{cc} 1/2 & 0 \end{array}\\ \begin{array}{cc} 1 & 0 \end{array} \end{array}\\ \begin{array}{c} 1/3\\ 0 \end{array} & \begin{array}{c} \begin{array}{cc} 1/3 & 1/3 \end{array}\\ \begin{array}{cc} 0 & 1 \end{array} \end{array} \end{array}\right]$$

$$A^{\text{Transpose}}=A_{\mathrm{DC}}=\left[\begin{array}{ccc} 1 & 1 & \begin{array}{ccc} 0 & 1 & 0 \end{array}\\ 0 & 1 & \begin{array}{ccc} 1 & 1 & 0 \end{array}\\ 0 & 0 & \begin{array}{ccc} 0 & 1 & 1 \end{array} \end{array}\right],T_{\mathrm{DC}}=\left[\begin{array}{ccc} 1/3 & 1/3 & \begin{array}{ccc} 0 & 1/3 & 0 \end{array}\\ 0 & 1/3 & \begin{array}{ccc} 1/3 & 1/3 & 0 \end{array}\\ 0 & 0 & \begin{array}{ccc} 0 & 1/2 & 1/2 \end{array} \end{array}\right]$$

Then the *HeteSim* score between *c*_*2*_ and *c*_*4*_ is:

HeteSimc2c4=TCD2:TDC4:TransposeTCD2:2TDC4:2=12,12,013,13,13Transpose12,12,0213,13,132=1316=63≈0.8165

#### 2.2.1 Disease semantic similarity

From MeSH database, each disease can be expressed as a directed acyclic graph (DAG), a disease $d_{i}$ can be represented as ${\mathrm{DAG}}_{d_{i}}=\left(d_{i},T_{d_{i}},E_{d_{i}}\right)$, where $T_{d_{i}}$ is the ancestor nodes of $d_{i}$ (including $d_{i}$ itself), $E_{d_{i}}$ is the set of corresponding edges, then the semantic contribution of a disease $d_{t}$ in ${\mathrm{DAG}}_{d_{i}}$ can be calculated by:

(5) $$\begin{array}{c} D_{d_{i}}\left(d_{t}\right)=\left\{\begin{array}{c} 1,i=t\\ \max\left\{∆*D_{d_{i}}\left(d_{t^{\prime }}\right)\right\},\text{else} \end{array}\right. \end{array}$$

∆ is the semantic contributor factor (from previous studies, we set ∆=0.5 here), then the semantic value of disease $d_{i}$ is defined as:

(6) $$\begin{array}{c} \mathrm{DV}\left(d_{i}\right)=\sum_{d_{t}\in T_{d_{i}}}D_{d_{i}}\left(d_{t}\right) \end{array}$$

and the semantic similarity between $d_{i}$ and $d_{j}$ is defined as:

(7) $$\begin{array}{c} \mathrm{SS}\left(d_{i},d_{j}\right)=\frac{\sum_{d_{t}\in T_{d_{i}}\cap T_{d_{j}}}\left(D_{d_{i}}\left(d_{t}\right)+D_{d_{j}}\left(d_{t}\right)\right)}{\mathrm{DV}\left(d_{i}\right)+\mathrm{DV}\left(d_{j}\right)} \end{array}$$

#### 2.2.2 Disease Gaussian Interaction Profile (GIP) kernel similarity

The GIP kernel similarity is widely used to measure the similarities among biomolecules, from HMDD v3.2 database, we can obtain the miRNA‐disease association matrix *MD*, each column of *MD* can be considered as the interaction profile of disease, given two diseases $d_{i}$ and $d_{j}$, the GIP kernel similarity between them can be calculated as follows:

(8) $$\begin{array}{c} \mathrm{GD}\left(d_{i},d_{j}\right)=\exp\left\{-\beta_{d}{\left\|\mathrm{MD}\left(:,i\right)-\mathrm{MD}\left(:,j\right)\right\|}^{2}\right\},\beta_{d}=\frac{n}{\sum_{i=1}^{n}{\left\|\mathrm{MD}\left(:,i\right)\right\|}^{2}} \end{array}$$

where *MD*(:,*i*) is the *i*‐th column of *MD*, *n* is the number of disease.

The disease similarity matrix ${\mathrm{SD}}^{\mathrm{bio}}$ is obtained by combining the semantic similarity and GIP kernel similarity, that is:

(9) $$\begin{array}{c} {\mathrm{SD}}^{\mathrm{bio}}\left(d_{i},d_{j}\right)=\left\{\begin{array}{c} \mathrm{SS}\left(d_{i},d_{j}\right),\mathrm{SS}\left(d_{i},d_{j}\right)\;\text{exists}\;\\ \mathrm{GD}\left(d_{i},d_{j}\right),\text{otherwise} \end{array}\right. \end{array}$$

#### 2.2.3 CircRNA expression profile similarity

exoRBase integrated RNA expression profile information based on normalized RNA‐seq data, for example, the expression profile information of circRNA $c_{i}$ can be expressed as $f_{i}=\left(f_{i1},f_{i2},\cdots ,f_{\mathrm{ih}}\right)$, and spearman correlation coefficient is used to measure the similarities among circRNAs.

(10) $$\begin{array}{c} \mathrm{SE}\left(c_{i},c_{j}\right)=1-\frac{6\sum d_{k}^{2}}{h\left(h^{2}-1\right)} \end{array}$$

where $d_{k}=f_{\mathrm{ik}}-f_{\mathrm{jk}}$ is the difference of rank, *h* is the dimension of feature vector.

#### 2.2.4 circRNA functional similarity

After obtaining the disease similarity matrix *SD*, we can define circRNA functional similarity as follows:

(11) $$\begin{array}{c} \mathrm{SF}\left(c_{i},c_{j}\right)=\frac{\sum_{t\in \left[1,u\right]}\mathrm{SD}\left(d_{\mathrm{it}},N\left(c_{j}\right)\right)+\sum_{t\in \left[1,v\right]}\mathrm{SD}\left(d_{\mathrm{jt}},N\left(c_{i}\right)\right)}{u+v},\\ \mathrm{SD}\left(d_{i},N\left(c_{i}\right)\right)=\max\mathrm{SD}\left(d_{i},d_{t}\right),t\in \left[1,\left|N\left(c_{i}\right)\right|\right] \end{array}$$

where $N\left(c_{i}\right)=\left\{d_{i1},d_{i2},\cdots ,d_{\mathrm{iu}}\right\}$ is the set of diseases‐related to circRNA $c_{i}$.

Finally, combine the expression profile similarity and functional similarity, we can obtain the circRNA similarity ${\mathrm{SC}}^{\mathrm{bio}}$:

(12) $$\begin{array}{c} {\mathrm{SC}}^{\mathrm{bio}}\left(c_{i},c_{j}\right)=\left\{\begin{array}{c} 0.5\times \left(\mathrm{SF}\left(c_{i},c_{j}\right)+\mathrm{SE}\left(c_{i},c_{j}\right)\right)\;\mathrm{SE}\left(c_{i},c_{j}\right)\;\text{exists}\;\\ \mathrm{SF}\left(c_{i},c_{j}\right),\text{otherwise} \end{array}\right. \end{array}$$

#### 2.2.5 Integrated similarity for circRNAs and diseases

The disease similarity and circRNA similarity are calculated as follows:

(13) $$\begin{array}{c} \begin{array}{c} \mathrm{SD}\left(d_{i},,,d_{j}\right)=0.5*{\mathrm{SD}}^{\mathrm{bio}}\left(d_{i},,,d_{j}\right)+0.25*\left(\text{HeteSim}\left(d_{i},,,d_{j}\right)\right.\\ \left.+\text{Pathsim}\left(d_{i},,,d_{j}\right)\right),i,j=1,2,\cdots ,n \end{array} \end{array}$$

(14) $$\begin{array}{c} \begin{array}{c} \mathrm{SC}\left(d_{i},,,d_{j}\right)=0.5*{\mathrm{SC}}^{\mathrm{bio}}\left(c_{i},,,c_{j}\right)+0.25*\left(\text{HeteSim}\left(c_{i},,,c_{j}\right)\right.\\ \left.+\text{Pathsim}\left(c_{i},,,c_{j}\right)\right),i,j=1,2,\cdots ,m \end{array} \end{array}$$

### 2.3 Methods

#### 2.3.1 Metapath‐based feature extractor

As shown in Figure 3(A), we selected eight different metapaths on circRNA‐miRNA‐disease heterogeneous network, however, the numbers of circRNAs, miRNAs and diseases are different, we apply a node type‐specific linear transformation layer here to project different types of nodes into same vector space, that is:

(15) $$\begin{array}{c} h_{c}^{\prime }=W_{c}∙S_{c},h_{d}^{\prime }=W_{d}∙S_{d} \end{array}$$

where $W_{c}\in {\mathbb{R}}^{m\times l}$, $W_{d}\in {\mathbb{R}}^{n\times l}$ are the weight matrices, $S_{c}\in {\mathbb{R}}^{m}$, $S_{d}\in {\mathbb{R}}^{n}$ are the original feature vectors of different types of nodes, here, we use the integrated similarity matrices of circRNAs and diseases as its original features, that is: $S_{c}\in {\mathbb{R}}^{m}$ is the *c*‐th row of *SC*, $S_{d}\in {\mathbb{R}}^{n}$ is the *d*‐th row of *SD*, *l* is the dimension of vector space.

A special metapath instance encoder is introduced here to transform the features of all nodes along the instance into a single vector:

(16) $$\begin{array}{c} h_{p\left(o_{1},o_{t}\right)}=f_{\theta }\left(o_{1},o_{t}\right)=f_{\theta }\left(h_{o_{1}}^{\prime },h_{o_{t}}^{\prime },\left\{h_{u}^{\prime },\forall u\in \left\{p\left(o_{1},o_{t}\right)/\left\{o_{1},o_{t}\right\}\right\}\right\}\right) \end{array}$$

where $p\left(o_{1},o_{t}\right)$ is a metapath instance connecting entities $o_{1}$ and $o_{t}$.

Then, multi‐head attention mechanism is used to aggregate instances under same metapath, the goal is learning the weight of each instance and the weighted summing of all instances is considered as the features of nodes.

(17) $$\begin{array}{c} e_{o_{1}o_{t}}^{p}=\text{LeakyReLU}\left(\alpha_{p}^{T}∙\left[h_{o_{1}}^{\prime }∥h_{p\left(o_{1,}o_{t}\right)}\right]\right),\alpha_{o_{1}o_{t}}^{p}=\text{softmax}\left(e_{o_{1}o_{t}}^{p}\right),\\ h_{o_{1}}^{P}={∥}_{k=1}^{K}\sigma \left(\sum_{u\in N_{o_{1}}^{p}}{\left[\alpha_{o_{1}o_{t}}^{p}\right]}_{k}∙h_{p\left(o_{1,}o_{t}\right)}\right) \end{array}$$

And the attention mechanism is also used to aggregate the information of different metapath as follows:

(18) $$\begin{array}{c} s_{p_{i}}=\frac{1}{\left|\Gamma_{v_{i}}\right|}\sum_{o_{1}\in \Gamma_{v_{i}}}\tanh\left(W_{v_{i}}∙h_{o_{1}}^{p_{i}}+b_{v_{i}}\right),e_{p_{i}}=q^{\text{Transpose}}∙s_{p_{i}},\\ \beta_{p_{i}}=\text{softmax}\left(e_{p_{i}}\right),h_{o_{1}}^{P}=\sum_{p_{i}\in P}\beta_{p_{i}}∙h_{o_{1}}^{p_{i}} \end{array}$$

where $v_{i}$, *i* = 1,2 corresponding to circRNA and disease nodes, $\Gamma_{v_{i}}$ is the node‐specific set, $\left|\Gamma_{v_{i}}\right|$ is the number of nodes in $\Gamma_{v_{i}}$, $p_{i}$ is the metapath instances related to node type *i*.

The objective function of metapath‐based feature extractor is defined as follows:

(19) $$\begin{array}{c} L=\frac{1}{N}\sum_{i}-\left(y_{\mathrm{ij}}∙\log\left(h_{c}^{\text{Transpose}}∙h_{d}\right)+\left(1-y_{\mathrm{ij}}\right)\log\left(1-h_{c}^{\text{Transpose}}∙h_{d}\right)\right) \end{array}$$

where *N* is the number of samples.

#### 2.3.2 Matrix factorization‐based feature extractor

Matrix factorization (MF) can project features of circRNAs and diseases onto same low‐dimensional vector space. As shown in Figure 3(B), the goal of MF is minimizing the following objective function:

(20) $$\begin{array}{c} H=CD^{\text{Transpose}}s.t.C\geq 0,D\geq 0,\mathrm{SC}=CC^{\text{Transpose}},\mathrm{SD}=DD^{\text{Transpose}} \end{array}$$

An indictor matrix *W* is introduced here, if there is a known association between circRNA and disease pair, $W_{\mathrm{ij}}=1$, else, $W_{\mathrm{ij}}=0$, then the objective function can be written as follows:

(21) $$\begin{array}{c} L=\arg\min_{C,D}{\left\|W∙\left(H-CD^{\text{Transpose}}\right)\right\|}_{F}^{2}+\alpha \left({\left\|C\right\|}_{F}^{2}+{\left\|D\right\|}_{F}^{2}\right)\\ +\lambda \left({\left\|\mathrm{SC}-CC^{\text{Transpose}}\right\|}_{F}^{2}+{\left\|DD^{\text{Transpose}}\right\|}_{F}^{2}\right) \end{array}$$

where *H* is the adjacency matrix of circRNA‐disease association network, C and D are the latent feature matrices of circRNAs and diseases, α and λ are the trade‐off parameters, ${\left\|∙\right\|}_{F}^{2}$ is the square of Frobenius norm, *SC* and *SD* are the similarity matrices of circRNAs and diseases, the alternating direction multiplier update rule is used here.

#### 2.3.3 GraphSAGE‐based feature extractor

Traditional graph convolutional networks (GCNs) update the node representations of the whole graph in each iteration, when the scale of the graph is large, the training strategy is undoubtedly time‐consuming and even can not be updated, this promotes researchers to introduce the idea of mini‐batch in GCN algorithms; therefore, GraphSAGE algorithm had been proposed.

The details of GraphSAGE algorithm can be summarized as follows:

1. Neighbour sampling: different from traditional GCN algorithms, GraphSAGE update the representation of the target node using the information of neighbours, specially, if the number of neighbours is greater than the pre‐defined number of samples, the oversampling (resampling) strategy is used, conversely, if the number of neighbours is less than the pre‐defined number of samples, the under‐sampling technique is used, which is shown in Figure 3 (c).
2. Aggregation: for simplicity, the mean aggregator is used in this study, that is:

(22) $$\begin{array}{c} h_{v}^{L}\leftarrow \sigma \left(W∙\text{MEAN}\left(h_{v}^{L-1}\cup h_{v}^{L-1},\forall u\in N\left(v\right)\right)\right) \end{array}$$

where $h_{v}^{0}$ is the original feature representation of node *v*, represented by the similarity matrices of circRNAs and diseases.

#### 2.3.4 Model fusion via ensemble learning

In order to obtain the optimal performance, the ensemble learning is used here, in this study, some classic classifiers are chosen, support vector machine (SVM),^38^ random forest (RF),^39^ extreme gradient boosting (XGBoost),^40^ light gradient boosting machine (LightGBM),^41^ gaussian naïve bayes (Gaussian NB),^42^ where RF is a variant of bagging, XGBoost and LightGBM are boosting algorithm. After obtain the classification results via different models and classifiers, a soft voting strategy is used to obtain the final predicted result of circRNA‐disease pair (Algorithm 1).

ALGORITHM 1 **Ensemble Learning based CircRNA‐Disease Association prediction (ELCDA)**

Input: circRNA‐disease association matrix *CD*; circRNA‐miRNA association matrix *CM*;

miRNA‐disease association matrix *MD*; circRNA and disease similarity matrices *SC*, *SD*;

the dimension of vector space *l*; the number of heads in metapath‐based feature extractor *K*;

the trade‐off parameter in MF‐based feature exactor *λ*;

1. Training the metapath‐based, MF‐based and GraphSAGE‐based feature extractors, obtaining the embeddings of nodes, denoted as *F*_*1*_, *F*_*2*_ and *F*_*3*_, respectively, and concatenate them as the final representations of nodes, denoted as $F=\text{concat}\left(F_{1},F_{2},F_{3}\right)$;

2. Using selected classifiers to obtain the predicted results;

3. Using soft voting strategy to obtain the final predicted results.

Output: the final predicted association probability $p\in \left(0,1\right)$.

## 3 EXPERIMENTS AND RESULTS

### 3.1 Evaluation metrics

To evaluate the performance of our model, we compared our propose model with other state‐of‐the‐art methods under fivefold cross‐validation (5‐cv). Specifically, the known circRNA‐disease associations in circR2Disease v2.0 is taken as the positive samples, and we randomly select negative samples with the same number of positive samples, and a balanced data set with 5940 samples can be obtain. The indicators to evaluate the model including *AUC* (the area under ROC curve), *AUPR* (the area under precision‐recall curve), *Accuracy*, *Recall* and *F*1‐score, we treat the association prediction as a binary classification problem, then the evaluate indicators can be defined as follows:

(23) $$\begin{array}{c} \text{Accuracy}=\frac{\mathrm{TP}+\mathrm{TN}}{\mathrm{TN}+\mathrm{TP}+\mathrm{FN}+\mathrm{FP}},\text{Recall}=\frac{\mathrm{TP}}{\mathrm{TP}+\mathrm{FN}},\\ \text{Precision}=\frac{\mathrm{TP}}{\mathrm{TP}+\mathrm{FP}},F1-\text{score}=\frac{2*\text{Precision}*\text{Recall}}{\text{Precision}+\text{Recall}} \end{array}$$

### 3.2 Parameters analysis

**FIGURE 4:**
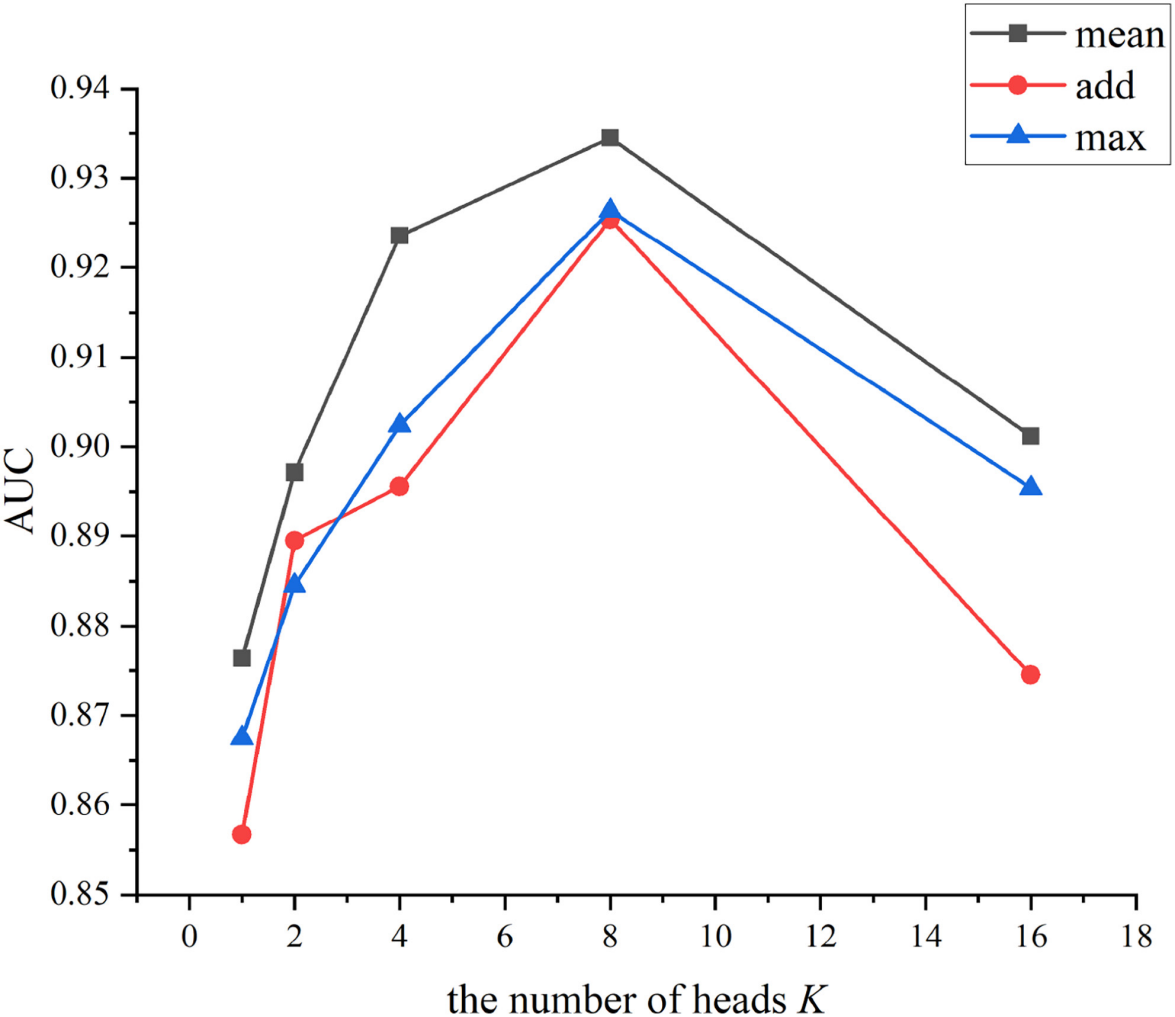
AUCs with different parameters combinations.

In this section, we analyse two main parameters of ELCDA, first, the number of heads *K* in MAGNN, second, the aggregator used in GraphSAGE, the results are shown in Figure 4, when *K* is 8 and the aggregator is *mean*, ELCDA obtains the best performance.

### 3.3 Comparison with other methods

In this paper, we compare ELCDA with seven other state‐of‐the‐art (SOTA) methods:

KATZHCDA (2018)^13^: predicting unknown associations based on KATZ measure;
CD‐LNLP (2019)^43^: predicting circRNA‐disease associations via linear neighbour label propagation;
KATZCPDA (2019)^44^: based on the original KATZHCDA model, taking into the impact of proteins to predict the associations between circRNAs and diseases;
icircDA‐MF (2020)^16^: predicting the potential disease‐associated circRNAs based on matrix factorization, and the circRNA‐disease interaction profiles are then updated by the neighbour interaction profiles so as to correct the false negative associations;
DMFCDA (2021)^45^: using deep matrix factorization to improve prediction of circRNA‐disease associations;
GMNN2CD (2022)^46^: using variational inference and graph Markov neural networks to predict circRNA‐disease associations;
AGAEMDA (2023)^47^: predicting unknown associations via node‐level attention graph auto‐encoder.

**FIGURE 5:**
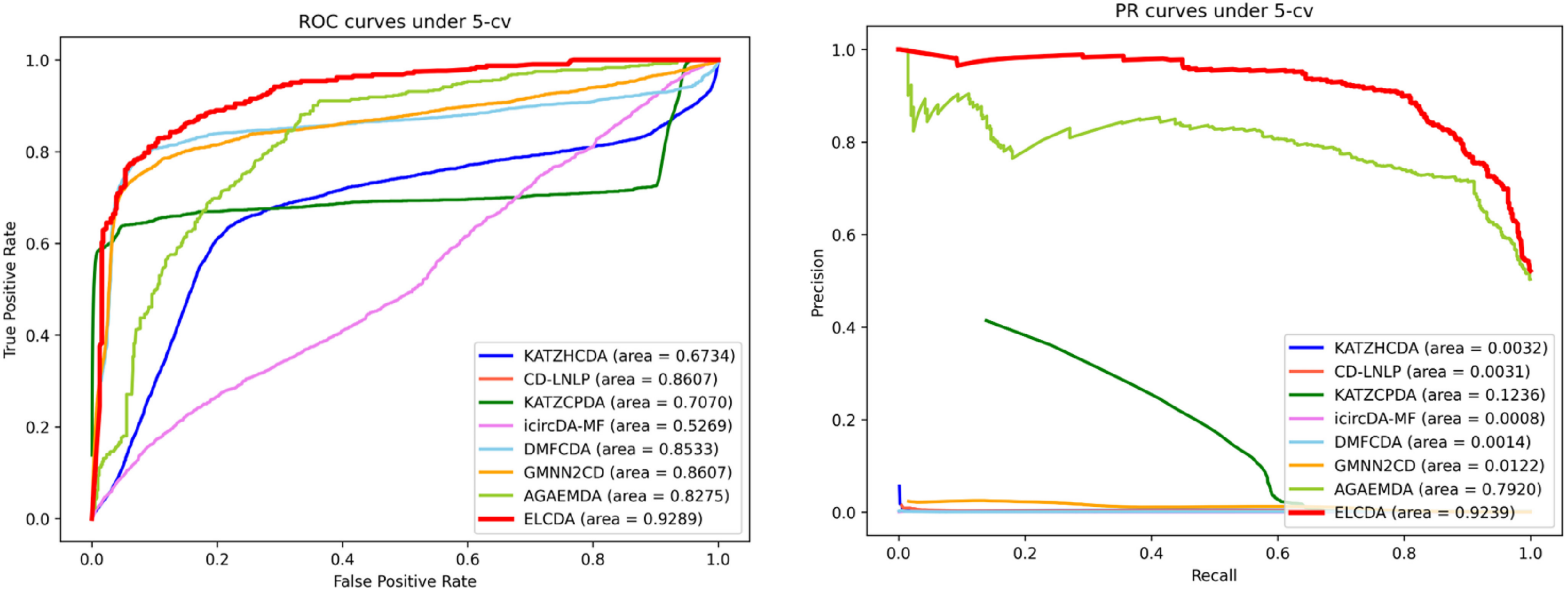
The ROC curves (left) and PR curves (right) under 5‐cv on different models.

The ROC, PR curves are shown in Figure 5. From which we can observe that our proposed ELCDA has the best performance under both AUC and AUPR, which achieves 0.9289 and 0.9239 under 5‐cv, outperforms all selected SOTA methods. Specifically, the AUPR values of KATZHCDA, CD‐LNLP, KATZHCPDA, icircDA‐MF, DMFCDA and GMNN2CD are significantly lower than AGAEMDA and ELCDA, cause the former methods didn't adopt any sample balance strategy, the ratio of positive and negative samples is close to 1:150, which indicates that the dataset used in this paper is extremely imbalanced, neglecting to perform data set balancing and preprocessing may lead to less‐than‐ideal results under AUPR and some other evaluation metrics. Other indicators are listed in Table 3, and the bold values are the maximums, from the results we can also observe that the performance of ELCDA is superior than other SOTA methods in most cases.

**TABLE 3:** The performance results of methods.

Method	Indicator		
	Accuracy	Recall	F1‐score
CD‐LNLP	0.498	0.717	0.005
KATZHCDA	0.523	**0.921**	0.013
KATZCPDA	0.499	0.804	0.010
icircDA‐MF	0.501	0.832	0.016
DMFCDA	0.501	0.832	0.016
GMNN2CD	0.500	0.862	0.005
AGAEMDA	0.774	0.911	0.795
ELCDA	**0.856**	0.832	**0.852**

*Note*: Bold value indicates the maximum value of each column.

### 3.4 Ablation studies

**FIGURE 6:**
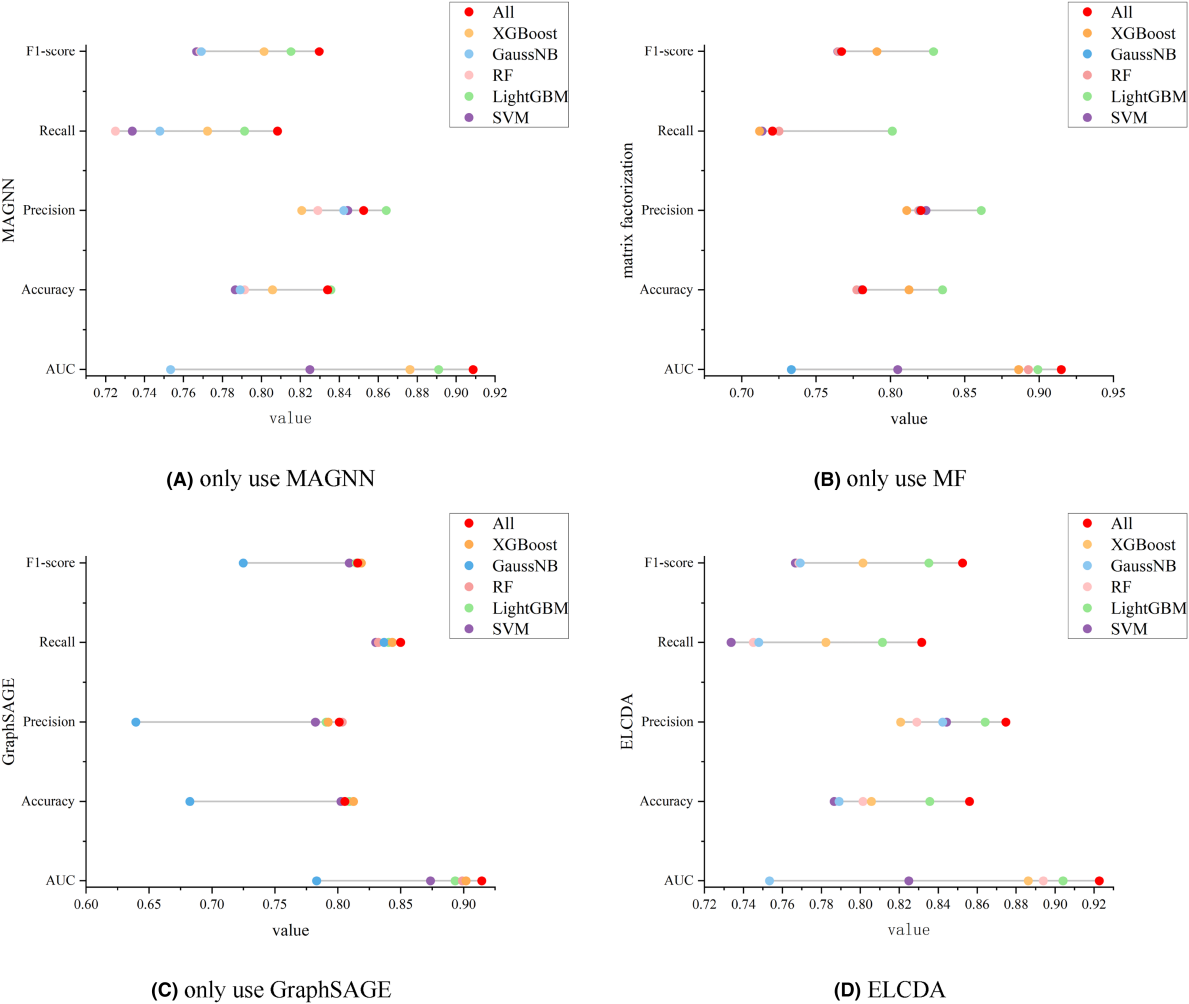
Performances on different module combinations. (‘All’ means all classifiers are used and soft voting adopted).

We use three feature extractors and various classifiers in this paper, the ablation studies are adopted here to illustrate the effectiveness of different module, the results are shown in Figure 6. It is obvious that our proposed model obtains the best performance on different metrics. Actually, SVM is a common and basic classifier, but not applicable to the case with lots of missing data; RF is a common bagging classifier, performs well in most cases, but overfitting may occur in noisy classification problems; XGBoost and LightGBM are variants of gradient boosting decision tree (GBDT) algorithm, which are faster and more robust, but not considering the concept that the optimal solution is a synthesis of all features; GaussianNB is a classifier based on naïve bayes, which is extremely fast, but performs poor of data with large size. Voting strategy is a classical ensemble learning algorithm, and compare with hard voting, soft voting strategy can achieve higher classification performance.

### 3.5 Case studies

Hepatocellular carcinoma (HCC), breast cancer (BC) and lung cancer (LC) are used to demonstrate the effectiveness of the proposed model.

At present, the global incidence of Liver Cancer is on the rise. It is estimated that by 2025, the annual number of liver cancer cases will exceed one million, where HCC is the most common type of LC, about 90% of the total number of cases.^48^ HCC is a prototypical inflammation‐associated cancer, which is the most common type of cancer among American adults, most patients have no symptoms in the early stages, HCC is a primary cancer that originates from hepatocytes in the extensively hardened liver tissue. Table 4 lists the top‐20 precited circRNAs related to HCC, and the results show that 20 of the top‐20 had been confirmed, demonstrates excellent predictive performance.

**TABLE 4:** Predicted top‐20 circRNAs related to HCC.

Rank	circRNA	Evidence	Rank	circRNA	Evidence
1	hsa_circ_100084	32,323,783	11	hsa_circ_0001001	32,222,024
2	hsa_circ_0008514	31,004,447	12	hsa_circ_0049613	32,393,764
3	hsa_circ_0139897	31,456,215	13	hsa_circ_0018764	30,675,276
4	hsa_circ_0010090	32,907,351	14	hsa_circ_0091570	31,207,319
5	hsa_circ_0110102	33,891,564	15	hsa_circ_0008717	32,196,586
6	circSMG1.72	31,996,784	16	hsa_circ_0001141	28,636,993
7	circZEB	30,123,094	17	hsa_circ_0008450	30,556,306
8	circWHSC1	33,410,156	18	hsa_circ_0003731	30,630,697
9	hsa_circ_0001175	33,408,482	19	hsa_circ_0000098	30,092,792
10	hsa_circ_0019456	31,417,632	20	circGFRA1	332,154,221

BC is a malignant tumour that occurs in the epithelial tissue of the breast gland, according to the latest report released by the International Agency for Research on Cancer (IARC) in 2022, it has become “the most common cancer in the world” with 2.26 million new cases worldwide. Several factors can increase the risk of developing it, include age, family history of BC (mainly related to gene mutations), obesity and so on. Early‐stage BC may not cause noticeable symptoms, the awareness and early detection campaigns can significantly improve the survival rates. Table 5 lists the predicted top‐20 circRNAs related to breast cancer, from which we can see that 20 of the top‐20 had been confirmed by biologists, which demonstrate ELCDA has the ability of giving the reliable candidate circRNA biomarkers of BC.

**TABLE 5:** Predicted top‐20 circRNAs related to BC.

Rank	circRNA	Evidence	Rank	circRNA	Evidence
1	circKLHL24	33,781,094	11	hsa_circ_0001283	32,066,649
2	hsa_circ_000911	29,431,182	12	circTADA2A	32,002,039
3	hsa_circ_0089105	30,657,346	13	hsa_circ_0064923	30,785,332
4	hsa_circ_100438	29,431,182	14	hsa_circ_0103021	30,979,827
5	circNINL	33,479,730	15	circYY1	33,603,460
6	hsa_circ_0125597	31,729,134	16	hsa_circ_406697	28,484,086
7	hsa_circ_0069094	31,943,203	17	hsa_circ_0064923	30,785,332
8	hsa_circ_0068515	33,136,699	18	hsa_circ_100219	31,127,997
9	circNR3C2	33,530,981	19	hsa_circ_0004619	28,484,086
10	hsa_circ_0100213	30,979,827	20	hsa_circ_0000002	30,810,051

LC is one of the most common and deadliest cancers all over the world, there are two main types of lung cancer, non‐small cell lung cancer and small cell lung cancer, and the former is more common, accounting for about 85% of all cases. Similar to BC, early diagnosis and treatment of it are crucial factors that can improve the chances of survival for individuals. The predicted top‐20 circRNAs related to LC are listed in Table 6, and 16 of them were confirmed via relevant researches, which can also show that ELCDA is an effective model for predicting circRNA‐disease associations.

**TABLE 6:** Predicted top‐20 circRNAs related to LC.

Rank	circRNA	Evidence	Rank	circRNA	Evidence
1	circHIPK3	30,352,682	11	hsa_circ_0007331	unconfirmed
2	hsa_circ_0067934	33,155,212	12	hsa_circ_101975	unconfirmed
3	circABCB10	32,420,810	13	circANKRD12	31,185,953
4	circITCH	27,642,589	14	hsa_circ_0072088	unconfirmed
5	circZNF609	33,459,380	15	circMAN1A2	31,046,163
6	hsa_circ_0001821	27,928,058	16	hsa_circ_0008193	31,700,878
7	hsa_circ_0008717	32,572,881	17	hsa_circ_0012673	32,141,553
8	hsa_circ_0014235	33,292,236	18	hsa_circ_0079471	unconfirmed
9	circBANP	29,969,631	19	circMAN2B2	29,550,475
10	hsa_circ_0046264	29,891,014	20	hsa_circ_0022812	32,511,866

**FIGURE 7:**
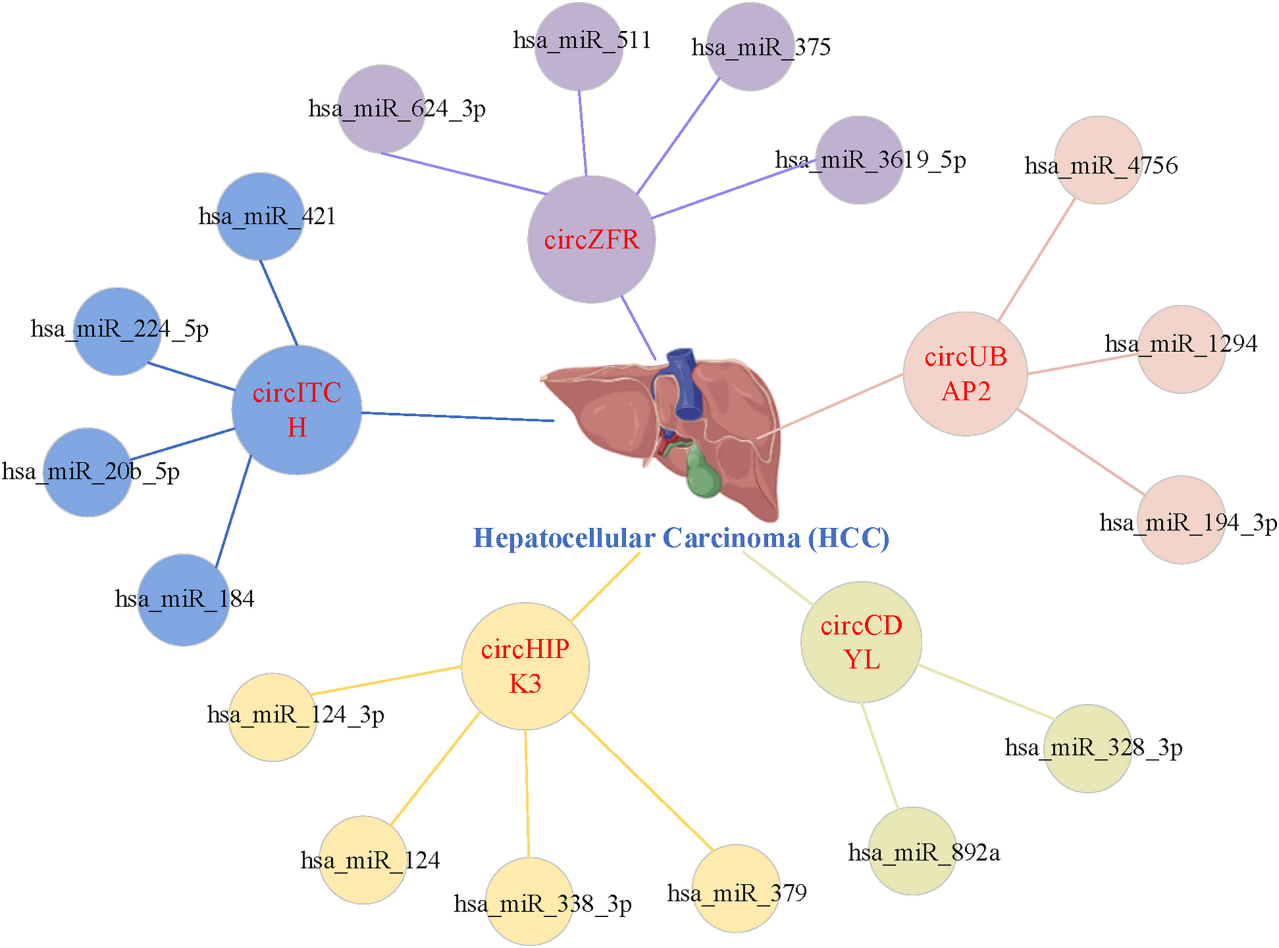
Example of ‘circRNA can act as the sponge of miRNA’.

Furthermore, taking HCC as an example, as shown in Figure 7, circITCH can act as the sponge of hsa‐miR‐184, hsa‐miR‐224‐5p, hsa‐miR‐20b‐5p and hsa‐miR‐421, which indicates one of the mechanisms of action of circRNAs: circRNA can function as miRNA sponges by binding to them and preventing their interactions with target mRNAs, thereby affecting the occurrence and development of human complex diseases; thus, circITCH may have an inhibitory effect on HCC.^49^ Researchers can work on the downstream mRNA and explore the potential role of circRNA by screening for the upstream circRNAs and identifying the corresponding circRNA‐miRNA‐mRNA pathway.

Actually, there are increasing researches focus on the interactions among non‐coding RNAs (ncRNAs) and other biological entities to better understand their regulatory mechanisms in diseases. For instance, circRNA‐miRNA,^50^, ^51^ miRNA‐lncRNA^52^, ^53^ and metabolite‐disease interaction predictions,^54^, ^55^ by investigating the interactions among ncRNAs, we can gain a better understanding their roles in cellular regulation and disease development. This knowledge can provide new insights and potential therapeutic targets for disease diagnosis and treatment. However, there are still many aspects that require further research to unravel the regulatory networks and mechanisms of ncRNAs.

## 4 CONCLUSIONS

With gradually deepening of researching, an increasing body of evidence suggests that circRNAs play a crucial role in the occurrence and development of human complex diseases, which can be regarded as biomarkers for diagnosis, treatment and prognosis. More and more studies have been conducted using the experimentally verified circRNA‐disease associations with computational models, and most existing methods ignore the information carried by the heterogeneous network and the intermediate nodes (miRNAs).

To address these drawbacks, in this paper, we propose an ensemble learning‐based model ELCDA for predicting circRNA‐disease associations. Compared with the previous models, the *HeteSim* and *PathSim* are introduced here to enhance the model for extracting information from heterogeneous, and circRNA‐miRNA, miRNA‐disease associations are given, with not only provide more detailed biological information, but also expand the variety of nodes. As the number of node types increasing, more kinds of metapaths can be defined. In addition, this study also adopts GraphSAGE and MF‐based feature extractor to obtain the comprehensive representations of nodes, and soft voting strategy is used to get the final predicted results. The results of numerous experiments indicate that ELCDA is outperforming than most SOTA models.

The proposed model still has shortcomings, which can be conducted in subsequent work in the further. First, the heterogeneous network can be constructed with different links, that is, other nodes can be introduced in the model, like gene, then circRNA‐gene, gen‐disease, gene–gene associations can be used to further enrich the biological information of circRNAs and diseases. Second, with more associations introduced, more kinds of metapaths can be selected, which will lead the model more effective and robust.

## AUTHOR CONTRIBUTIONS

**Jing Yang:** Conceptualization (equal); data curation (equal); formal analysis (equal); methodology (lead); validation (equal); visualization (equal); writing – original draft (equal). **Xiujuan Lei:** Resources (supporting); supervision (lead); writing – review and editing (equal). **Fa Zhang:** Writing – review and editing (equal).

## FUNDING INFORMATION

This work was supported by the National Natural Science Foundation of China under Grand (Nos. 62272288) and the Fundamental Research Funds for the Central Universities, Shaanxi Normal University (GK202302006).

## CONFLICT OF INTEREST STATEMENT

The authors confirm that there are no conflicts of interest.

## Data Availability

The data that support the findings of this study are openly available in [circR2Disease V2.0]/[https://doi.org/10.1016/j.gpb.2021.10.002.], reference number [9].
